# Enhancement of the Solubility and Bioavailability of Pitavastatin through a Self-Nanoemulsifying Drug Delivery System (SNEDDS)

**DOI:** 10.3390/pharmaceutics14030482

**Published:** 2022-02-22

**Authors:** Mehran Ashfaq, Shahid Shah, Akhtar Rasul, Muhammad Hanif, Hafeez Ullah Khan, Ahmed Khames, Mohamed A. Abdelgawad, Mohammed M. Ghoneim, Muhammad Yasir Ali, Mohammad A. S. Abourehab, Safirah Maheen, Omeira Iqbal, Ghulam Abbas, Amani M. El Sisi

**Affiliations:** 1 Department of Pharmaceutics, Faculty of Pharmaceutical Sciences, Government College University, Faisalabad 38000, Pakistan; mehran.ashfaq@pkli.org.pk (M.A.); akhtar.rasul@gcuf.edu.pk (A.R.); myasirali@gcuf.edu.pk (M.Y.A.); pharmacist@carepharmacy.com.pk (O.I.); 2 Department of Pharmacy Practice, Faculty of Pharmaceutical Sciences, Government College University, Faisalabad 38000, Pakistan; 3 Department of Pharmaceutics, Faculty of Pharmacy, Bahauddin Zakariya University, Multan 60000, Pakistan; muhammad.hanif@bzu.edu.pk; 4 Department of Pharmaceutics, College of Pharmacy, University of Sargodha, Sargodha 40100, Pakistan; hafeezullah.khan@uos.edu.pk (H.U.K.); safirah.maheen@uos.edu.pk (S.M.); 5 Department of Pharmaceutics and Industrial Pharmacy, College of Pharmacy, Taif University, Taif 21944, Saudi Arabia; a.khamies@tu.edu.sa; 6 Department of Pharmaceutical Chemistry, College of Pharmacy, Jouf University, Sakaka 72341, Saudi Arabia; mhmdgwd@ju.edu.sa; 7 Department of Pharmacy Practice, Faculty of Pharmacy, AlMaarefa University, Ad Diriyah 13713, Saudi Arabia; mghoneim@mcst.edu.sa; 8 Department of Pharmaceutics, Faculty of Pharmacy, Umm Al-Qura University, Makkah 21955, Saudi Arabia; maabourehab@uqu.edu.sa; 9 Department of Pharmaceutics and Industrial Pharmacy, College of Pharmacy, Minia University, Minia 61519, Egypt; 10 Department of Pharmaceutics and Industrial Pharmacy, Faculty of Pharmacy, Beni-Suef University, Beni-Suef 62514, Egypt; amany.elsese@pharma.bsu.edu.eg

**Keywords:** nanoemulsion, pitavastatin, drug release, cytotoxicity, pharmacokinetics

## Abstract

The purpose of the study was to develop an SNEDDS to improve the solubility and bioavailability of pitavastatin. The solubility of pitavastatin in different oils, surfactants, and co-surfactants was determined and a pseudo-ternary phase diagram was constructed. The SNEDDS was characterized by zeta-sizer, zeta-potential, FTIR, DSC, and TGA. Release and permeation of pitavastatin from the SNEDDS was studied for 12 and 24 h, respectively. The lipolysis test, RBC lysis, effect on lipid profile, and pharmacokinetics were studied. The SPC3 formulation showed a 104 ± 1.50 nm particle size, a 0.198 polydispersity index (PDI), and a –29 zeta potential. FTIR, DSC, and TGA showed the chemical compatibility and thermal stability. The release and permeation of pitavastatin from SPC3 was 88.5 ± 2.5% and 96%, respectively. In the lipolysis test, the digestion of SPC3 yielded a high amount of pitavastatin and showed little RBC lysis. The lipid profile suggested that after 35 days of administration of the SNEDDS, there was a marked decrease in TC, LDL, and triglyceride levels. The SNEDDS of SPC3 showed an 86% viability of Caco-2 cells. Pharmacokinetics of SPC3 showed improved values of C_max_, T_max_, half-life, MRT, AUC, and AUMC compared to the reference formulation. Our study demonstrated that the SNEDDS effectively enhanced the solubility and bioavailability of a BCS class II drug.

## 1. Introduction

Hyperlipidemia is a condition in which there are increased amounts of lipids in the blood. Modern primary care physicians spend a lot of time and money on preventive medicine. Diagnosis and treatment of hyperlipidemia as a means of preventing cardiovascular disease is a common practice of primary care physicians [1]. After hypertension, hyperlipidemia ranks as the second leading condition among the 10 most common chronic conditions observed, according to data from the Centers for Disease Control from a study of 1492 physicians [2]. Several clinical trials have shown that statins successfully lower low-density lipoprotein and lower cardiovascular danger in individuals with dyslipidemia and metabolic disorder [3].

The oral route is the most common, efficient, profitable, and feasible route for administration. Although it has several disadvantages, such as the first pass effect, low bioavailability, low stability, it is the most adoptive approach to administer the drug due to the high rate of patient compliance [4]. Pitavastatin, which belongs to a class of statins, is a synthetic inhibitor of HMG-CoA reductase that has a cyclopropyl group. It is indicated to minimize elevated levels of total cholesterol (TC), low-density lipoprotein cholesterol (LDL-C), apo-lipoprotein B (Apo B), triglycerides (TGs), and improve high-density lipoprotein cholesterol (HDL-C) in the treatment of adult patients with primary hyperlipidemia or mixed dyslipidemia. It is also recommended for the care of pediatric patients 8 years of age and older with heterozygous familial hypercholesterolemia (HeFH) to minimize elevated TC, LDL-C, and Apo B [5]. It has the same effects as other statins, that is, pravastatin, atorvastatin, and simvastatin but at a very low dose [6].

Pitavastatin belongs to the BCS class II; therefore, it has low solubility and bioavailability. It is metabolized in the liver by glucuronidation and is excreted through urine and feces. Because of this, bypassing the liver metabolism and improving the solubility of pitavastatin is an attractive approach to enhancing its therapeutic effect. Different approaches have been taken for the delivery of poorly soluble drugs, e.g., solid dispersion, particle size reduction, co-precipitation, and lipid-based drug delivery systems [7,8].

A lipid-based or lipid-carrier drug delivery system is a promising technique that has been used to improve the oral bioavailability of less soluble drugs. The primary goal of the lipid-based formulation is to minimize liver degradation and increase the bioavailability of drugs [9]. A self-emulsifying drug delivery system (SEDDS) is one of the lipid-based drug delivery systems currently being studied for its advantages: it provides a wide interfacial region to separate the drug between oil and gastrointestinal fluid. This technique increases the oral bioavailability of poorly soluble drugs by increasing the solubility and preserving the drug in a dissolved state, in small oil droplets, during its passage through the gastrointestinal tract [10]. In an SEDDS, when the emulsion droplet size is in the nanometer range, this formulation can be called an SNEDDS.

The SNEDDS has been more systematically characterized from a physicochemical point of view among lipid-based formulations. SNEDDS are an isotropic mixture of oil, surfactant, and co-surfactant with gentle agitation, which after interaction with water, join together to form an emulsion. An SNEDDS normally produces droplet sizes in the range of 20–200 nm after dilution. The prepared nano-sized droplets can provide improved dissolution rates as well as bioavailability. SNEDDS are generally more mechanically and physically stable compared to other conventional preparations (emulsion) and easy to prepare both on a small and large scale. The rationale for using SNEDDS for the delivery of less soluble drugs is that they can be obtained as a pre-concentrated solution, which will avoid the dissolution step. Furthermore, the development of various colloidal species in the dispersal and subsequent digestion of the SNEDDS promotes drug absorption [11,12]. The present study aimed to develop an SNEDDS of pitavastatin in order to enhance its solubility and bioavailability.

## 2. Materials and Methods

### 2.1. Materials

Pitavastatin was obtained as a gift sample from CCL Laboratories Pvt., Ltd., Lahore, Pakistan. PEG 200, PEG 400, PEG 600, cinnamon oil, olive oil, ginger oil, sesame oil, virgin coconut oil, and castor oil were purchased from Sigma–Aldrich Gmbh, Darmstadt, Germany. Methanol, acetonitrile, Tween 20, Tween 40, Tween 60, and Tween 80 were acquired from Merck, Darmstadt, Germany. Triton X-100 was obtained from Daejung Chemicals, Korea. Sodium hydroxide, glacial acetic acid, and potassium dihydrogen phosphate were obtained from Daejung Chemicals, Korea. Dimethyl sulfoxide (DMSO) and 4-bromophenylboronic acid were purchased from Merck, Darmstadt, Germany.

### 2.2. Solubility Study

The solubility of pitavastatin in different oils, surfactants, and co-surfactants was determined using a cyclomixer. Four milligrams of drug were added to 3 mL of each surfactant, co-surfactant, and essential oil. The mixture containing the drug was mixed by a cyclomixer for 24 h at 25 °C with 2000 rpm. The sample containing the drug was then removed from the mixer and centrifuged at 9000 rpm for 10 min to remove the amount of undissolved drug. The supernatant solution was then obtained from the mixture and passed through a 0.45 µm Millipore membrane filter and diluted with dimethyl sulfoxide. The amount of pitavastatin in the mixture was determined using a UV spectrophotometer at 245 nm [13]. The experiment was repeated in triplicate.

### 2.3. Construction of the Pseudo-Ternary Phase Diagram

The pseudo-ternary phase diagram was constructed using oils, surfactants, and co-surfactants. Based on solubility studies of pitavastatin, the oils, surfactants, and co-surfactants were selected. With the concentration of oil (20–60%), surfactant (30–80%), and co-surfactant (0–40%), a number of self-emulsifying series were prepared at 25 °C. Oils, surfactants, and co-surfactants mixtures with different concentrations were prepared in order to determine the self-emulsifying region. In the absence of pitavastatin, a pseudo-ternary phase diagram was constructed using CHEMIX^®^ software [14,15]. All observations were reported and repeated in triplicate.

### 2.4. Preparation of the Pitavastatin SNEDDS

After identifying the self-emulsifying region, the different ratios of oils, surfactants, and co-surfactants for the SNEDDS were selected for the incorporation of pitavastatin. Four milligrams of pitavastatin were weighed and mixed with cinnamon oil followed by the addition of Tween 80 and PEG 400 as shown in Table 1 [16]. After, the preparation was placed in a sonicator to obtain a clear solution. The prepared SNEDDS was then stored for further studies.

SPC1, 2, and 3 were SNEDDS of pitavastatin containing cinnamon oil; SPT1, 2, and 3 were SNEDDS of pitavastatin containing tea tree oil; and SPS1, 2, and 3 were SNEDDS of pitavastatin containing sesame oil.

### 2.5. Characterization of the SNEDDS

#### 2.5.1. Self-Emulsification Time

The time taken by the pre-concentrate to form a homogeneous mixture after which the SNEDDS visually disappears is known as the self-emulsification time. The self-emulsification times were determined by a dissolution apparatus (USP Apparatus II) with a paddle speed of 100 rpm at 37 ± 5 °C. Two microliters of the prepared SNEDDS were added dropwise to the medium (i.e., 900 mL of phosphate buffer) and the time until disappearance was calculated [17].

#### 2.5.2. Droplet Size Determination

The average droplet size was investigated by PCM (phase contrast microscope). For this purpose, 2 mL of the prepared SNEDDS were diluted 50, 100, and 500 times with water and phosphate buffer with pH 1.2, 3.0, and 6.8, and the effect of the dispersion medium and volume of the medium on the droplet size was comparatively studied [18].

#### 2.5.3. Particle Size Analysis

The average particle size of the prepared SNEDDS was investigated using a Malvern Zeta Sizer Nano Series ZS90. The polydispersity index indicates the uniformity of the particle diameter, and it may be used to determine the nanoemulsion’s size distribution. The sensitivity range of the Malvern Zeta Sizer was in between the range of 10 and 5000 nm. The temperature, while performing all these parameters, was maintained at 25 °C with an angle of 90° [19].

#### 2.5.4. Zeta Potential Analysis

The stability of the nanoemulsion depends on the surface charge. The zeta potential of the prepared SNEDDS was determined using the Malvern Zeta Sizer Nano Series ZS90. Before the analysis, the sample was diluted with distilled water in the ratio of 1:100 (*v*/*v*). The experiment was repeated in triplicate [20].

#### 2.5.5. Fourier Transform Infrared Spectroscopy (FTIR)

The FTIR spectra of pitavastatin, cinnamon oil, Tween 80, PEG 400, and unloaded and loaded drug formulations were analyzed by an FTIR spectrophotometer (Bruker Alpha, Germany) with a wavelength range of 400–4000 cm^−1^, and each sample was tested at 10 scans within the wavelength range.

#### 2.5.6. Differential Scanning Calorimetry (DSC)

For DSC, 5 ± 0.5 mg samples of pitavastatin, cinnamon oil, Tween 80, PEG 400, and unloaded and loaded drug formulations were placed in aluminum crucibles and heated from 50 to 400 °C at a 5 °C/min heating rate under a stream of nitrogen gas flow at 40 mL/min using a differential scanning calorimeter (DSC-60, Shimadzu, Germany).

#### 2.5.7. Thermogravimetric Analysis (TGA)

For TGA, 10 ± 0.5 mg sample of pitavastatin, cinnamon oil, Tween 80, PEG 400, and unloaded and loaded drug formulations were placed in aluminum crucibles and heated from 50 to 400 °C at a 10 °C/min heating rate under a stream of nitrogen gas flow at 40 mL/min. The heating temperature was considered to be the function of the percentage weight loss.

### 2.6. In Vitro Dissolution Studies

The in vitro drug release studies were performed using the dialysis bag method using a USP Apparatus II. The prepared SNEDDS of pitavastatin were added to the dialysis bag, which was then sealed. The sealed bag added in 900 mL of dissolution medium (phosphate buffer) with a pH of 6.8 at 37 °C. The speed of the paddle was maintained at 100 rpm [21]. With predetermined time intervals, 5 mL of sample was collected, and 5 mL buffer was added to maintain the sink condition. The release of pitavastatin from the prepared SNEDDS was compared with marketed formulations. A UV spectrophotometer determined the presence of drug content from the sample. All observations were reported and repeated in triplicate.

### 2.7. Kinetics of Pitavastatin Release

To obtain the release kinetics and evaluate the mechanism of pitavastatin release, the data on in vitro pitavastatin release from the SNEDDS were fitted to five mainly applied mathematical models (i.e., Korsmeyer–Peppas model, Hixon–Crowel model, Higuchi model, first order, and zero order) for the evaluation of the release kinetics of pitavastatin from the prepared SNEDDS.

### 2.8. Ex Vivo Permeation Studies

Adult Male Wistar rats, with a weight of 200–250 g, were kept in a room with a constant room temperature of 25 °C and relative humidity of 55% with free access to a normal meal and water. The rats were starved overnight but given water only before the experiment. Animals were sacrificed by dislocating their spines. By cutting between the lower end of the ileum and the upper end of the duodenum and removing the mesentery, the small intestine was directly separated followed by sacrifice. Using a syringe with a blunt end, the small intestine was thoroughly cleaned out with KRPB (Kreb’s Ringer phosphate buffer) solution. The intestinal sacs were cleaned and split into 6 cm pieces. One microliter of KPRB was used to distribute the selected SNEDDS. At the same drug concentration, a suspension of marketed pitavastatin tablet and pitavastatin powder (control) was also dispersed in 1 mL of KRPB. Six sacs were filled with the SNEDDS and TC solution (equal to 4 mg of the drug) using a blunt needle, while the other six sacs were filled with an equivalent amount of tablet and powder suspensions. A thread was used to tie the two sides of the intestine. Each sac was put into a glass test tube with 10 mL of KRPB solution. In a shaking water bath spinning at 100 rpm, the entire system was kept at 37 °C and aerated with a laboratory aerator. Over a period of 2 h, samples were removed from the outside of the sac and the medium was completely replaced with fresh medium. HPLC was used to evaluate the samples [22]. Pitavastatin permeability was determined by charting the total amount of medication penetrated through the sac verses time. The pitavastatin apparent permeability coefficient (PAAP) was derived using the following equation:

(1) $$PAPP\left(\frac{cm}{sec}\right)=\frac{\frac{dQ}{dt}}{\frac{A}{C_{0}}}$$

where *dQ*/*dt* is the drug permeation rate from the membrane, a is the cross-sectional area of the tissues, and *C*_0_ is the initial pitavastatin concentration in the donor compartment at *t*_0_.

### 2.9. In Vitro Lipolysis Tests

In the lipolysis test [23], the selected SNEDDS formulations were distributed in 9 mL of digestion buffer under fed (pH 5.0, 144 mM glacial acetic acid, 101 mM NaOH, and 203 Mm NaCl) and fasted (pH 6.5, 28.6 mM NaH_2_PO_4_.H_2_O, 10.5 mM NaOH, and 105.9 mM NaCl) conditions. In the digestion mixture, SIF powder comprising lecithin (phospholipid) and taurocholate (bile salt) in 1:4 molar ratios (the ratio secreted in bile) was incorporated. Prior to enzyme addition, the lipid formulations in the mixed micellar solutions were emulsified in the thermostatic jacketed glass reaction vessel by spinning constantly for 10 min. Trials were carried out at 37 °C, and to start the process of lipolysis, 1 mL of pancreatin extract containing 800 units of tributyrin of pancreatic lipase was added. Using a pH-stat titration machine that kept the pH constant at 6.8, lipolysis was allowed to continue for 30 min. All of the formulations had their fatty acids generated during lipolysis titrated with 0.2 M NaOH. Three samples of 2 mL digestion solution were transferred into polyallomer centrifuge tubes at the end of each digestion experiment, and 20 µL of 4-bromophenylboronic acid was added to each sample to inhibit further lipolysis. To separate the different digestion phases, the samples were ultra-centrifuged at 8000 rpm for 50 min at 4 °C using an SW-60 swinging bucket rotor. The formulation digests were separated into two phases after ultracentrifugation: aqueous and precipitated pellet. HPLC was used to determine the drug content of the samples obtained from each of the separated phases.

### 2.10. RBC Lysis Test

An RBC lysis test was performed for the evaluation of in vitro acute toxicity of the pitavastatin-loaded SNEDDS. Briefly, blood from a healthy human was placed in an anticoagulant solution and centrifuged at 1000× *g* for 15 min at 4 °C. The buffy coat and plasma-containing supernatant was discarded. The RBC-containing pellet was washed and diluted in isotonic buffer (150 mM NaCl and 10 mM phosphate buffer) to obtain a 50% hematocrit. The RBC suspension of aliquots were treated for 1 h at 37 °C with 4 mg/mL of each tested formulation. The aliquots were centrifuged at 1500× *g* for 1 h, after which the supernatant was analyzed for released hemoglobin with the help of UV Visible spectroscopy at 576 nm [23].

### 2.11. Effect of the SNEDDS Formulations on Lipid Profile

For this purpose, 24 rabbits were selected and divided into four groups and given favorable conditions. All groups had full access to diet and water. To induce hypercholesteremia, 100 mg/kg/day of cholesterol was administered to the rabbits for 15 days. The fasting cholesterol level was evaluated to ensure the induction of hypercholesteremia in rabbits. A parallel study design method was used to determine the effect of pitavastatin-containing SNEEDS on the rabbits’ lipid profile [24]. Group I was the control group, group II was the disease control, group III was the standard (treatment with marketed formulation Pitalo^®^), and group IV received an SNEDDS of pitavastatin for five weeks. Blood samples were collected and analyzed.

### 2.12. Stability Studies

The stability study was conducted by placing the SPC3 formulation in tightly sealed bottles for 90 days at a 45 ± 2.0 °C temperature and 75 ± 5.0% humidity. The samples were withdrawn at 0, 30, 60, and 90 days and analyzed for color, self-emulsification time, particle size, and zeta potential.

### 2.13. Cytotoxicity Studies

The Caco-2 model was used to evaluate the toxicity of the SNEDDS in the MTT assay for cell viability study. The ATCC (American Type Culture Collection) was the main source of the Caco-2 cell line. Eagles’ MEM (minimum essential medium) along with 20% FBS (fetal bovine serum) were mainly used for the cultivation of the Caco-2 cell in 96-well plates. Prior to treatment, all excipients (essential oil, Tween 80, and PEG 400), drug (pitavastatin), and formulations were diluted in DMEM (Dulbecco’s modified Eagle’s medium) with 0.5% DMSO. DMEM without FBS was used for incubation with cells after treatment with 200 µg/mL SPC3 and blank formulations and with the same concentration of excipients used in the SPC3 formulation. Following incubation, the samples were collected at a predetermined time, and the collected sample was washed with PBS. After, a solution of MTT was added to the medium having no FBS, and this preparation was incubated for 1 h and the supernatant removed and dissolved in DMSO. At a wavelength of 570 nm, the absorbance of the prepared solution was noted. The following equation was used to evaluate the percentage of cell viability [25]:

(2) $$Cell\;viability\;\left(\%\right)=\frac{As}{Ad}\times 100$$

where *A_s_* is absorbance after treatment with the sample dispersion, and *A_d_* is the absorbance after treatment with DMEM.

### 2.14. Pharmacokinetics of Pitavastatin

Two groups of six albino rats, a test group, and a control group, weighing between 0.3 and 0.5 kg, were selected for the pharmacokinetic studies. The pharmacokinetics evaluation was evaluated with the approval of the Ethical Committee for the Utilization of Laboratory Animals, Government College, University Faisalabad, Punjab, Pakistan. The ICH (International Council for Harmonization) rules and regulations were abided for the handling of albino rats while performing pharmacokinetic evaluation. The albino rats had full access to water and diet with a circadian cycle of light and dark. The temperature was maintained at 25 ± 5 °C for the entire period. The albino rats were abstained for 24 h prior to beginning the pharmacokinetic analysis, yet they were permitted free access to water. A feeding tube was used to administer the prepared SNEDDS and suspension of pitavastatin to the groups. The pitavastatin suspension and the SPC3 formulation were prepared in a 0.25% carboxymethyl cellulose solution and administered orally at a dose of 10 mg/kg. The volume administered was 5 mL. All rats were labeled accurately and confined in a wooden box while performing the collection of the samples. At a predetermined time period, a total of 0.25 mL blood was extracted from the tail of the rat and shifted to a centrifugation tube. The tube was centrifuged at 6000 rpm for 10 min and the plasma collected and stored at −20 °C. A liquid–liquid extraction method was used to obtain the pitavastatin from the plasma. The obtained sample was then reconstituted with 100 μL of the mobile phase and an HPLC-UV spectrophotometer was used to evaluate the pharmacokinetics of the prepared SNEDDS [26].

#### 2.14.1. Data Analysis

The concentrations of pitavastatin in the SNEDDS and in the suspension of the pitavastatin were evaluated by calibration curves, which were constructed using Microsoft Excel 2007. Kinetica R version 4.1.0 (Thermo Electron Corporation, USA) was used to evaluate the pharmacokinetics parameter. Equation (3) was used for the determination of the maximum concentration of the drug in plasma, *C_max_* ng/mL. Equation (4) was used to determine the time to reach the peak plasma concentration in blood, *t_max_*. The area under the plasma concentration from the time curve to time t, *AUC*_0-*t*_ ng/mL h, was determined using Equation (5). In addition, Equation (5) was used for the area under the plasma concentration from the time curve to infinity (*AUC*_0–∞_, ng/mL.h). Equation (6) was used for the determination of the area under the maximum concentration (AUMC). Clearance, Cl h^−1^ half-life, kel h^−1^, and mean residence time (*MRT*) were evaluated using Equation (7).

(3) $$C_{max}=\frac{{FX}_{0}}{V_{d}}\times {\;e}^{-{kt}_{max}}$$

(4) $$t_{max}=2.303\log(K_{a}/K_{e})\left(K_{a}-K_{e}\right)$$

(5) $$AUC_{0-t}=\overset{n}{\underset{1}{\sum }}\frac{C_{i}+C_{i}+1}{2}\cdot \Delta t$$

(6) $$AUMC_{0-t}=\overset{n}{\underset{1}{\sum }}\frac{\;t_{i}(C_{i}+C_{i}+1)}{2}\cdot \Delta t$$

(7) $$MRT=\frac{AUMC}{AUC}$$

where *F* is the dose fraction, *V_d_* is the volume of distribution, *K_a_* is the absorption rate constant, *K_e_* is the elimination rate constant, Δ*t* (*t*_2_–*t*_1_) is the time interval, *C_i_* is the Initial amount, and *C_i_*+1 is the final amount.

#### 2.14.2. Statistical Analysis

SPSS (SPSS Inc., Chicago, IL, USA) version 17 was used to evaluate the pharmacokinetic parameters statistically. The Student’s *t*-test was used to compare the results between the test group and control group.

## 3. Results and Discussion

### 3.1. Solubility Study

**Figure 1:**
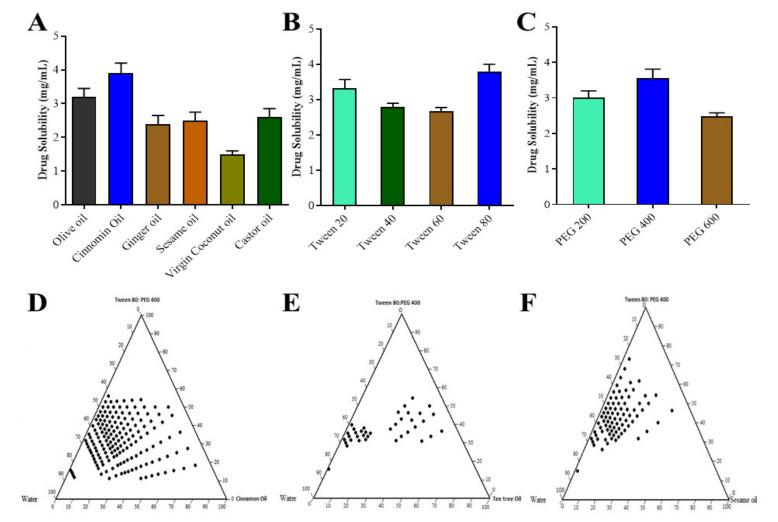
Solubility studies of pitavastatin in oils (**A**), surfactants (**B**), and co-surfactants (**C**); pseudo ternary phase diagram in cinnamon oil (**D**), tea tree oil (**E**) and sesame oil (**F**).

The solubility of pitavastatin in different excipients used in the formulation of the SNEDDS was evaluated in order to generate a stable region for self-emulsification in a ternary phase diagram with a large border size and a nano droplet size [27]. The pitavastatin solubility in various excipients is depicted in Figure 1A–C. Pitavastatin was found to have the highest solubility in cinnamon oil (3.9 mg/mL) and olive oil (3.2 mg/mL) and the lowest solubility in virgin coconut oil. Because of their propensity to solubilize pitavastatin, cinnamon oil, olive oil, ginger oil and castor oil were chosen as oil phases for future analysis. Tween 80 (3.8 mg/mL) and Tween 20 (3.32 mg/mL) had the maximum solubility of Pitavastatin among the several surfactants tested. Tween 80, a non-ionic surfactant, was used as a surfactant for this study, since it has been shown to improve medication absorption. PEG 400 (3.56 mg/mL) and PEG 200 (3.00 mg/mL) were shown to have good solubilization properties for pitavastatin. Based on their statistical significance, the abovementioned excipients were chosen for future studies.

### 3.2. Pseudo Ternary Phase Diagram

In the absence of pitavastatin, pseudo ternary phase diagrams, as shown in Figure 1D–F, were constructed to find the region of self-emulsification and optimize the percentages of a variety of excipients for the optimal formulation. Solubility analysis showed that olive oil, cinnamon oil, ginger oil, and castor oil had the maximum capability to solubilize the drug therefore these oils were used as oil phases. For generating multiple phase diagrams in order to determine the optimal self-emulsification region, PEG 400 and Tween 80 were used as co surfactants and surfactants, respectively. Turbid emulsions were produced when surfactant concentrations were 40–50%, which was significant. As the amount of co-surfactant in the SNEDDS was raised, the potential to spontaneously generate an emulsion inside self-emulsification zone improved. For self-emulsification, the minimal ratio of Smix was 45–75%. The effectiveness of self-emulsification of the SNEDDS formulation was improved when the Smix ratio (mixture of surfactant and co-surfactant) was greater than 60%. In order to generate a phase diagram, the effect of surfactants and co-surfactants on droplet size were assessed, and it was observed that droplet size was lowered from 270 to 75 nm as the surfactant concentration was increased from 30% to 60%. However, increasing the surfactant concentration to 70% caused the larger mean droplet size. The best self-emulsifying properties were discovered in nine different formulations [28].

### 3.3. Characterization of SNEDDS

#### 3.3.1. Self-Emulsification Time

The emulsification time was the most important indicator for the efficiency of self-emulsification. SNEDSS should disperse quickly and completely. Prepared SNEDDS formulation SPC3 showed a very less emulsification time, i.e., less than 1 min when compared to other prepared formulations.

#### 3.3.2. Droplet Size Analysis

The distribution of droplet size is one of the most significant emulsion characteristics for stability assessment and a key step in improving the bioavailability of drugs [29]. Prepared formulations SPC1, SPC2, and SPC3 were selected for droplet size analysis; the smaller the particle size leads to an enhanced the surface area, thus improving the absorption of the drug. The droplet size of all formulations was less than 200 nm. Among these formulations, SPC3 has the smallest droplet size, i.e., 109 ± 0.51 nm.

#### 3.3.3. Particle Size Distribution

**Figure 2:**
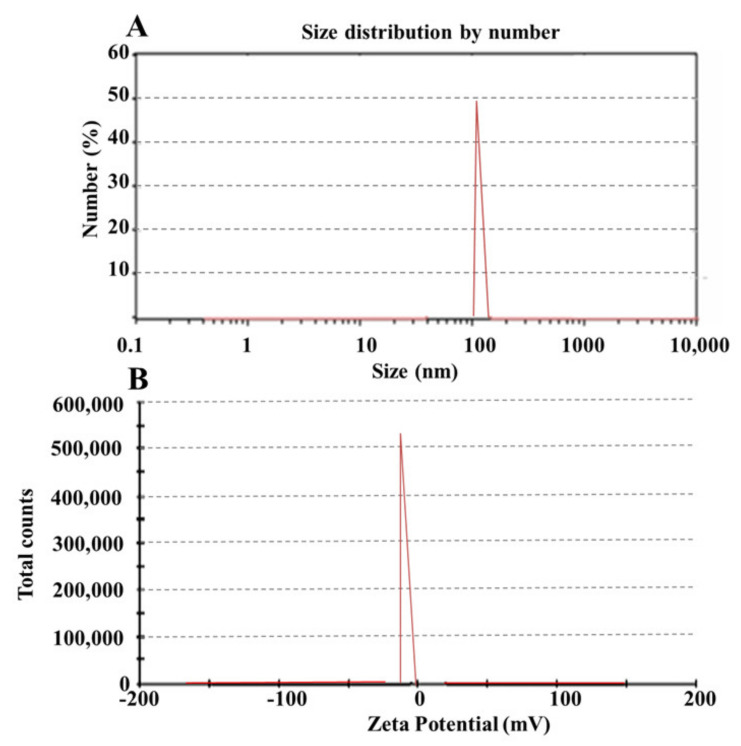
Zeta sizer (**A**) and zeta potential (**B**) of the SPC3 formulation.

The particle size distribution was assessed using the prepared formulations that had been chosen. Formulation having a smaller particle size leads towards rapid absorption and, thus, improved bioavailability [30]. From the selected formulations, SPC3 showed 104 ± 1.50 nm particle sizes as shown in Table 2A Figure 2A and the polydispersity index (PDI) of the SPC3 formulations was 0.198. The results indicated that when the oil concentration in the formulation increased, the particle size and PDI increased, and the zeta potential value changed from more negative to less negative as shown in Table 2.

#### 3.3.4. Zeta Potential

The prepared SNEDDS with the code names SPC1, SPC2, and SPC3 were selected for zeta potential analysis. The stability of the formulation depends on the zeta potential as it opposes aggregation [30]. Zeta potentials of the prepared formulation SPC1, SPC2, and SPC3 were –13, –16, and –29 as shown in Figure 2B. From these formulations SPC3 was the most stable one. A high negative charge on prepared formulation depends on the presence of free fatty acids. The value of zeta potential ±30 mV was enough for the system’s stability.

#### 3.3.5. Fourier Transforms Infrared Spectroscopy (FTIR)

**Figure 3:**
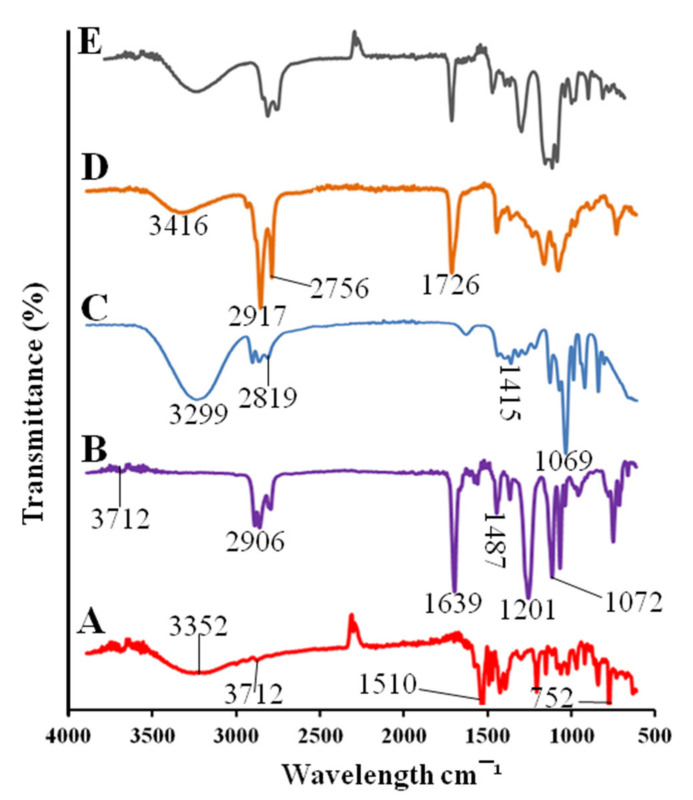
FTIR spectra of pitavastatin (**A**), cinnamon oil (**B**), PEG 400 (**C**), Tween 80 (**D**), drug-loaded SPC3 formulation (**E**).

The pitavastatin showed a peak of hydroxyl group at 3352 cm^−1^. The peak at 2912, 1510, and 752 cm^−1^ indicated the presence of C–H, carboxyl and C–F groups [31] as shown in Figure 3. The cinnamon oil showed peaks at 3712, 2904, 1639, 1487, 1201, and 1072 cm^−1^ were due to the amine and phenols, alkanes, ketone, ester and alkenes, nitro group, ether and esters [32] respectively. The spectra of PEG 400 showed O–H stretching at 3299 cm^−1^, C–H stretching at 2819 cm^−1^, C–H bending at 1415 cm^−1^, and C–O–H stretching was observed at 1069 cm^−1^ [33]. Tween 80 showed a stretching vibration of the hydroxyl group at 3416 cm^−1^. The asymmetric and symmetric stretching bonds of Tween 80 were observed at 2917 and 2756 cm^−1^, respectively, as shown in Figure 3. The stretching of C=O ester group was shown at 1726 cm^−1^ [34]. The drug-loaded formulation showed the peaks of drug, surfactants, and co-surfactants that indicated the chemical compatibility of the SNEDDS formulation.

#### 3.3.6. Differential Scanning Calorimetry (DSC)

**Figure 4:**
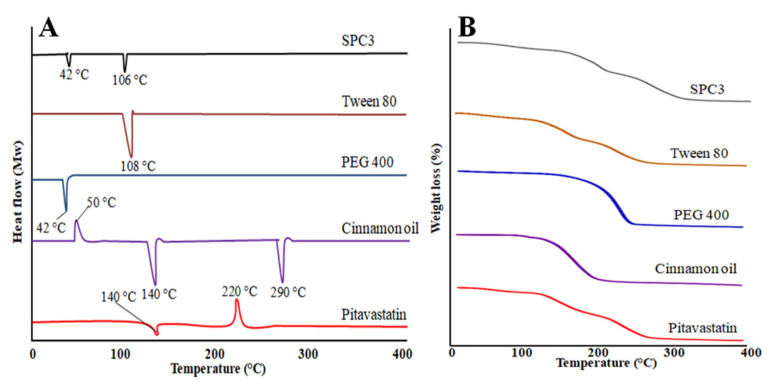
DSC thermograms (**A**) and TGA (**B**) curves of pitavastatin, cinnamon oil, PEG 400, Tween 80, and the SPC3 formulation.

Pitavastatin drug showed an endothermic peak at 140 °C and an exothermic peak at 220 °C as shown in Figure 4A. Cinnamon oil showed at 50 °C an exothermic peak and two endothermic peaks at 140 and at 290 °C. PEG 400 showed an endothermic peak at 42 °C and Tween 80 showed an endothermic peak at 108 °C. Our prepared SNEDDS formulation showed two endothermic peaks at 48 and 106 °C, and there was no peak of pitavastatin which indicates that drug was uniformly distributed in prepared SNEDDS formulation [35].

#### 3.3.7. Thermogravimetric Analysis (TGA)

Thermogravimetric analysis was used to evaluate the thermal stability of prepared SNEDDS formulation, cinnamon oil, PEG 400, Tween 80 and pitavastatin drug. Pitavastatin drug showed a weight loss starts from 100 to 300 °C and it was observed that after 300 °C there was no weight loss observed as shown in Figure 4B. Cinnamon oil showed a rapid weight loss starting from 100 to 200 °C. PEG 400 showed a speedy weight loss starting from 181 to 284 °C. Tween 80 showed a gradual weight loss starting from 141 to 296 °C. The prepared SPC3 SNEDDS formulation showed a gradual weight loss starting from 100 to 300 °C indicated the stability of SNEDDS [35,36].

### 3.4. In Vitro Drug Release Study and Release Kinetics

**Figure 5:**
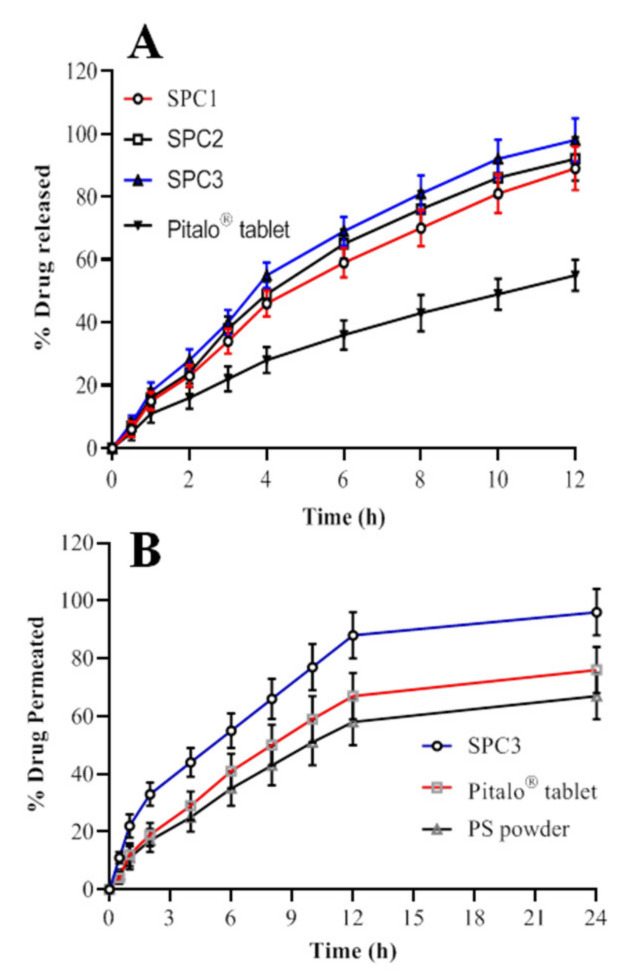
%age drug release (**A**) and %age drug permeated (**B**) of pitavastatin from the SNEDDS formulation.

Using 900 mL of dissolution medium with a pH of 6.8 at 37 °C, the in vitro drug release study was performed. The release pattern of pitavastatin from the SNEDDS showed that maximum release of the drug was observed with SPC3 formulation after 12 h as shown in Figure 5A. This could be due to the proper oil and surfactant composition of the system. The formulation of SPC1 showed a lower release of around 89.78 ± 0.88% of the drug, which may be due to a higher proportion of oil resulting in a larger droplet size and smaller surface area exposed to the medium [23]. It is evident from the data that the release rate of pitavastatin from the SNEDDS formulations was substantially higher than the commercially available drug formulation. The percentage release of pitavastatin from SPC3 was 98.5 ± 2.5% after 12 h, which was substantially higher than the marketed formulation, i.e., 55.45 ± 1.55%. Therefore, the in vitro findings reveal that the prepared SNEDDS formulation showed improved release of pitavastatin. The values of kinetics of pitavastatin release are shown in Table 3. The SNEDDS of pitavastatin showed the zero order kinetics with non-fickian diffusion release mechanism.

### 3.5. Ex Vivo Permeation Studies

The permeation studies were conducted to evaluate the in vitro release and prediction of in vivo absorption of Pitavastatin. The ex vivo intestinal permeation of pitavastatin from SNEDDS of SPC3 was evaluated and compared with pitavastatin powder (control) and commercialized tablet (Pitalo) by non-everted sac method [37] as shown in Figure 5B. The average plasma concentration of pitavastatin over a period of 24 hr period is shown in Figure 5B. The optimized SNEDDS (SPC3) formulation had the highest capability to permeate through the intestine as compared to the pitavastatin powder and Pitalo^®^. After 2 h the intestinal permeation of the optimized SPC3 SNEDDS reached 96%, Pitalo^®^ 76% and pitavastatin powder 67% as shown in Figure 5B. The drug is soluble in the permeation media and no solid particles were seen on the sac. The enhanced intestinal absorption of pitavastatin from SNEDDS formulation could be explained by number of ways. The quick permeation of pitavastatin inside the intestinal sac and quick diffusion could explain the high penetration. The enhanced drug absorption is because of the nano sized droplets of the emulsion in the region of intestine, which could improve the pitavastatin absorption, also nanoemulsion provide a greater surface area for drug penetration through intestine [22]. Furthermore, the SPC3 formulation have high drug solubility along with rapid self-emulsification may have aided pitavastatin absorption in the intestine. The bio-enhancing capability of the surfactant (Tween 80) and co-surfactants (PEG 400) improves the permeability by disrupting cell membrane lipids [38]. In vitro drug release investigation showed a similar correlation.

### 3.6. Lipolysis Test

**Figure 6:**
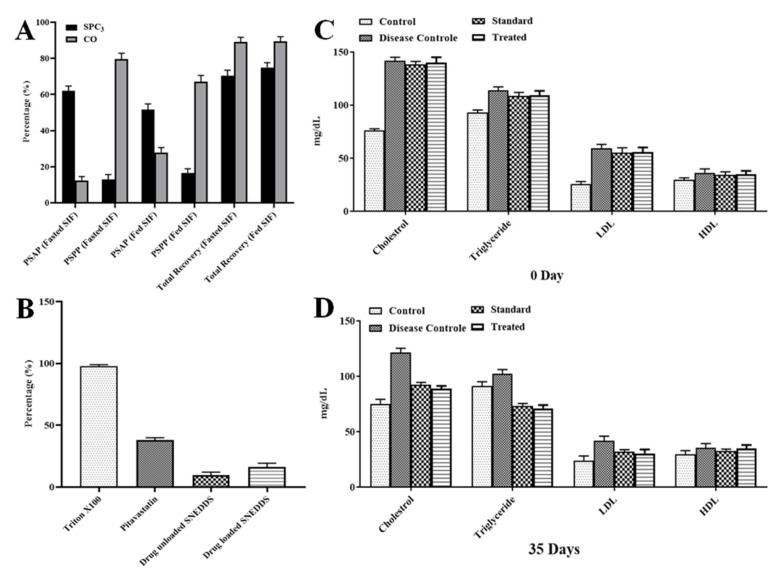
In vitro lipolysis (**A**), RBC lysis (**B**) and lipid profile of albino rats at 0 (**C**) and 35 days (**D**). CO, is cinnamon oil (pitavastatin in oil); PSAF, pitavastatin in aqueous phase; PSPP, pitavastatin in pellet phase; SIF, simulated intestinal fluid; LDL, low-density lipoprotein; HDL, high-density lipoprotein.

Digestion of the SNEDDS in the small intestine is important for the release of the incorporated drug. Digestion of SNEDDS in aqueous phase release a significant amount of drug as compared to pellet phase suggesting greater permeability of drug through intestine. The values of percentage of drug recovery were also adjusted. This test was carried out to verify the in vivo behavior of the SNEDDS. The SPC3 formulation was selected to check the digestion in fasted and fed conditions and results are shown in Figure 6A. Under fasting condition, the aqueous and pellet phase showed 62.45% and 13.23% digestion of the SPC3 formulation and 12.39% and 83.74% digestion of the TC formulation. In the fasted condition, the total recovery of PS from SPC3 and CO after 30 min was 74.34% and 89.17%, respectively. In the fed condition, the aqueous and the pellet phase showed a 52.15% and 17.73% digestion of SPC3 formulation and 28.19% and 67.24% digestion of TC formulation. In the fed condition, the total recovery of PS from SPC3 and CO after 30 min was 75.18% and 90.30%, respectively. In the aqueous phase, the digestion of SPC3 yielded a high amount of PS compared to the CO formulation. CO had greater solubility of PS in the pellet phase and retained a good quantity of PS [39,40]. The in vitro lipolysis test demonstrated the bio-relevance for the release and absorption of PS from the lipid-based SNEDDS formulation. In the lipolysis test, the quantity of drug is determined form the SNEDDS when the lipolysis reaction was completed.

### 3.7. RBC Lysis Test

An in vitro RBC lysis test was performed to see if the SNEDDS formulation caused RBC toxicity. The use of Triton X-100 as a positive control resulted in 100% RBC lysis [23]. The RBC lysis caused by the optimized SNEDDS. The pitavastatin-loaded SNEDDS generated very little RBC lysis as compared to pure pitavastatin powder and blank SNEDDS formulation as shown in Figure 6B.

### 3.8. Effect of the SNEDDS Formulation on Lipid Profile

Pitavastatin at 4 mg/kg body weight for five weeks reduced low density lipoproteins (LDL-C). Total cholesterol and triglycerides by 54%, 38%, and 28%, respectively, in rats fed a fatty mixed diet. The level of HDL-C did not alter considerably [23]. Cinnamon’s significant lipolytic action may play a direct role in lipid metabolism, preventing hypercholesterolemia and hypertriglyceridemia and lowering free fatty acids. Cinnamon oil reduces hepatic cholesterol levels by inhibiting HMG Co-A reductase activity and suppressing lipid peroxidation by increasing hepatic antioxidant enzyme activity [41] as shown in Figure 6C,D.

### 3.9. Stability Studies

There was no change in color was observed but a significant (*p* < 0.05) change in self emulsification time was observed. The article size of SPC3 formulation was 106 ± 2.45, 109 ± 2.21, and 113 ± 2.14 nm at 30, 60 and 90 days, respectively. The value of zeta potential was –28, –25, and –21 at 30, 60, and 90 days, respectively.

### 3.10. CytotoxicityS

**Figure 7:**
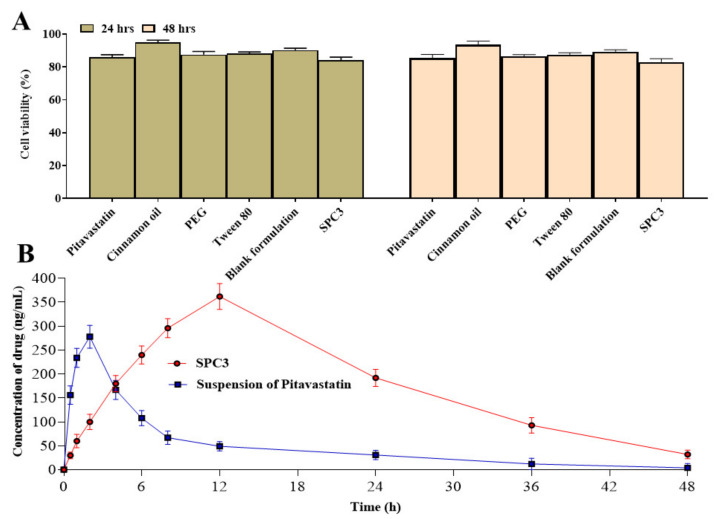
Cytotoxicity study of drug, excipients, blank formulation and SPC3 (**A**) and pharmacokinetic analysis of pitavastatin from SPC3 (**B**).

We examined the viability of caco-2 cell cultures treated with pitavastatin, cinnamon oil, PEG 400, Tween 80, and SPC3 formulation. The results showed that SPC3 in culture medium did not affect the viability of these cells at the dose used in the study. The cell viability plot showed that more than 86% cells were viable after 48 h of incubation with SPC3 formulation as shown in Figure 7A. No alteration of cell morphology of the cell lines was observed under the microscope.

### 3.11. Pharmacokinetic Analysis

Table 4 shows the pharmacokinetic properties of pitavastatin based on the plasma concentration versus time profile. The reference formulation (suspension of pitavastatin) had a *C_max_* of 278 ± 3.609 ng/mL, while the SPC3 SNEDDS had a *C_max_* of 362 ± 4.786 ng/mL (*p* < 0.05). The SPC3 SNEDDS and suspension of pitavastatin had *T_max_* of 12 ± 1.543 h and 2 ± 0.543 h (*p* = 0.002), respectively as shown in Figure 7B. The SPC3 SNEDDS had a greater AUC0-∞ 9338 ± 9.876 ng/mL.h (*p* = 0.001) than the suspension of pitavastatin (2478 ± 6.409 ng/mL.h). The MRT of SPC3 SNEDDS and suspension of pitavastatin were 22.49 ± 2.345 and 13.09 ± 1.059 h, respectively. The greater MRT value suggested that the drug in the SPC3 SNEDDS formulation was released more slowly than the suspension of pitavastatin. When compared to suspension of pitavastatin, the SPC3 SNEDDS had a greater relative bioavailability. To summarize, when comparing drug retention duration in the blood, *C_max_*, and AUC values, SPC3 SNEDDS provides better in vivo behavior than suspension of pitavastatin.

## 4. Conclusions

Finally, based on pitavastatin solubility data, we produced several self-emulsifying formulations. Cinnamon oil (40%), Tween 80 (40%), and PEG 400 (20%) were shown to have the optimum self-emulsification properties for pitavastatin. It was observed that the particle size and droplets size of the optimized SPC3 SNEDDS were 104 ± 1.50 nm and 109 ± 0.51, respectively, which improved the compound’s solubility. The zeta potential criterion for stability was met by the improved formulation. In-house repeatable and reliable HPLC method was developed for the measurement of Pitavastatin in rat plasma. In vivo investigation showed that the optimized pitavastatin SNEDDS had a greater *C_max_* (*p* = 0.003) and AUC (*p* = 0.001) than the reference formulation (pitavastatin suspension), indicating a significant improvement in bioavailability. Efficient digestion of SNEDDS in the presence of bile salts enhances drug release, which could improve drug penetration through the intestinal mucosa, thereby improving drug bioavailability. Moreover, the observed plasma time concentration in rat plasma analyzed with Kinetica provides a comprehensive pharmacokinetic investigation of pitavastatin SNEDDS and is the best fit for the one compartment model. Lastly, when the pitavastatin SNEDDS were compared to the marketed formulation (pitavastatin suspension), the prepared SNEDDS had greater bioavailability. This could be due to the fact of a collective mechanism including nano-sized dispersion with a larger surface area, which results in improved drug diffusion, enhanced drug solubility, lymphatic bypass share and mucosal permeability.

## Figures and Tables

**Table 1:** Formulations for SNEDDS of pitavastatin.

Formulation Code	Pitavastatin (mg)	Essential Oils (%)	Tween 80 (%)	PEG 400 (%)
SPC1	4	30	60	10
SPC2	4	50	30	20
SPC3	4	40	40	20
SPT1	4	30	60	10
SPT2	4	50	30	20
SPT3	4	40	40	20
SPS1	4	30	60	10
SPS2	4	50	30	20
SPS3	4	40	40	20

**Table 2:** Particle size and zeta potential of developed formulations.

Formulation Code	Particle Size (nm)	PDI	Zeta Potential
SPC1	112 ± 2.02	0.208	−16
SPC2	116 ± 1.31	0.338	−13
SPC3	104 ± 1.50	0.198	−29
SPT1	124 ± 2.12	0.372	−17
SPT2	119 ± 2.10	0.467	−15
SPT3	111 ± 1.27	0.301	−25
SPS1	114 ± 2.05	0.408	−27
SPS2	121 ± 1.10	0.467	−17
SPS3	108 ± 1.01	0.351	−20

**Table 3:** Values of kinetics of the pitavastatin release.

Code	Korsemeyer-Peppas		Hixon–Crowell	Higuchi	First-Order	Zero-Order
	*R^2^*	*n*	*R^2^*	*R^2^*	*R^2^*	*R^2^*
SPC1	0.9980	0.829	0.9968	0.9140	0.9826	0.9939
SPC2	0.9974	0.885	0.9949	0.8942	0.9880	0.9917
SPC3	0.9982	0.789	0.9961	0.9280	0.9927	0.9995
SPT1	0.9940	0.706	0.9979	0.9506	0.9939	0.9995
SPT2	0.9902	0.661	0.9978	0.9607	0.9879	0.9997
SPT3	0.9898	0.681	0.9986	0.9540	0.9729	0.9817
SPS1	0.9938	0.671	0.9904	0.9613	0.9857	0.9913
SPS2	0.9971	0.700	0.9927	0.9561	0.9758	0.9903
SPS3	0.9985	0.657	0.9909	0.9706	0.9834	0.9979

**Table 4:** Parameters of pharmacokinetics of SPC3 and suspension of pitavastatin.

Parameters	SPC3 (Test Formulation)	Suspension of Pitavastatin (Reference Formulation)
*t_max_* (h)	12 ± 1.543	2 ± 0.543
*C_max_* (ng/mL)	362 ± 4.786	278 ± 3.609
*t*_1/2_ (h)	12.24 ± 2.345	2.12 ± 0.678
*AUC*_0-*t*_ (ng/mL.h)	7696 ± 12.789	2257 ± 5.785
*AUC*_0-∞_ (ng/mL.h)	9338 ± 9.876	2478 ± 6.409
*AUMC*_0-∞_ (ng/mL.h)	210,039 ± 20.897	60,598 ± 10.762
*MRT* (h)	22.49 ± 2.345	13.09 ± 1.059

## Data Availability

Not applicable.
